# Radiation exposure and estimated risk of radiation-induced cancer from thoracic and abdominal radiographs in 1307 neonates

**DOI:** 10.1007/s00330-024-10942-x

**Published:** 2024-07-16

**Authors:** Deliah Weiß, Martin Beeres, Ulrich Rochwalsky, Thomas J. Vogl, Rolf Schlößer

**Affiliations:** 1 Paediatrics and Adolescent Medicine/Department of Neonatology, Clinic of the Goethe University, Frankfurt, Germany; 2 Institute for Diagnostic and Interventional Radiology, Clinic of the Goethe University, Frankfurt, Germany; 3 Clinic for Neuroradiology, Marburg University Hospital, Marburg, Germany

**Keywords:** Infant, Newborn, Intensive care, Diagnostic imaging, Radiation

## Abstract

**Objective:**

This study examined radiation exposure and the possible risk of radiation-induced cancer in a large sample of newborn and premature patients.

**Material and methods:**

In this retrospective study, we included all hospitalised neonates treated at our university hospital who received at least one X-ray examination from 1 January 2013 to 31 December 2018. We evaluated the dose area product (DAP), effective dose (ED), and estimated risk. The International Commission on Radiological Protection Publication 60 defines values (2.8–13 × 10^−2 ^Sv^−1^) to calculate the estimated risk in relation to the ED.

**Results:**

Of the 3843 patients (aged 241.1 ± 35.45 days) treated in the neonatal care unit, 1307 (34%) received at least one X-ray. The mean number of X-ray examinations per patient was 3.19 and correlated negatively with birth weight. The mean cumulative DAP was 5.9 mGy*cm^2^, and the cumulative ED was 23.7 µSv per hospital stay. Patients with a birth weight of < 1000 g showed the highest cumulative ED and DAP (*p* < 0.001). Patients with a birth weight of < 2500 g had the highest ED and DAP per image (*p* < 0.001). The highest radiation exposure (ED/DAP) occurred for thoracic/abdominal examinations, especially for neonates < 500 g (*p* < 0.001).

**Conclusion:**

There is a strong correlation between immaturity, the number of X-ray examinations, and radiation exposure. The total exposure was minimal, and the number of X-rays per patient has been decreasing in recent years.

**Clinical relevance:**

Possible risks to newborns and premature infants caused by ionising X-rays are often the subject of scientific and clinical discussion. Nevertheless, conventional X-ray imaging remains a frequently used tool, and total exposure remains at a very low level.

**Key Points:**

- *The number of X-rays per patient has been decreasing in a large university hospital*.
- *Half of all patients received only one X-ray; most had a birth weight over 1500* *g*.
- *This radiation risk can be classified as ‘minimal’ for patients with a birth weight of <* *500* *g and as ‘negligible’ for others*.

## Introduction

In the field of neonatology, patients are often born with serious internal and/or surgical conditions or are at risk of certain complications resulting from their prematurity. Diseases of the respiratory tract and the lungs remain a major threat to mature, sick newborns as well as premature infants. In the acute clinical setting, these infants may be subjected to a large number of diagnostic procedures and therapies within a short period of time. Conventional X-ray imaging remains an important tool in the acute medicine context to gain sufficient diagnostic information or to follow-up on the results of a given treatment. In addition, X-ray imaging can be combined with ultrasound, which is not using ionising radiation [1–6]. Patients who are treated in a neonatal intensive care unit (NICU) are only a few days old and therefore are undergoing active cellular proliferation and have a high number of undifferentiated cells. In some cases, many examinations as well as images have to be performed in a NICU. The goal is for these patients to survive the neonatal period so that they may live a long life. Therefore, X-ray exposure during early life remains an important issue. Moreover, in the context of computed tomography (CT), researchers have reported a positive correlation between the applied radiation dose and the occurrence of childhood leukaemia as well as brain tumours [7–9]. Taken together, the effect of radiation exposure in children, especially in preterm newborns, should not be underrated.

In this retrospective study, we analysed X-ray exposure based on the number of X-ray examinations, the X-ray technique and the dose used. Specifically, we examined:

- The frequency of X-rays in relation to birth weight and age, and how the trend has changed over time;
- The relation between immaturity and the type of imaging;
- The relation between radiation exposure and birth weight, the type of imaging and the cumulative dose; and
- The relation between the immaturity of neonates and the severity of the disease and the cumulative radiation dose.

## Material and methods

This retrospective, single-centre study was approved by the local institutional review board, which waived the requirement for written informed consent. All procedures were carried out according to the national ethical standards and the 1964 Declaration of Helsinki and its subsequent amendments.

Image analyses were performed on a dedicated workstation using the local picture archiving and communications system (GE Centricity Universal Viewer, GE Healthcare). A radiology resident (W.D., with three years of experience in paediatric imaging) and a paediatric radiologist (B.M., with seven years of experience in paediatric imaging) evaluated the images retrospectively. Each individual reviewed the images independently.

### Patients

Frankfurt University Children’s Hospital has a level 1 NICU (the highest level in Germany). It has 12 beds; in addition, there are two attached intermediate care units with 24 beds in total. In the NICU, 500–700 patients are treated each year. In this study, the clinical data from 1307 patients who were treated in the NICU between 1 January 2013 and 31 December 2018 were analysed retrospectively. The patients had to have received at least one X-ray examination to be included. Patients who died within the first 14 days (*n* = 32) and patients whose medical records could not be traced (*n* = 17) were excluded.

### Data collection

The data were collected from the local patient data management systems, namely ORBIS® (Agfa Healthcare), Enaio® (Optimal Systems) and GE Centricity Universal Viewer (GE Healthcare). The collected data included the dose area product (DAP) in µGy*m^2^, the current-time product in mAs, the tube voltage in kV, the type of examination (thoracic, thoracic/abdominal or abdominal), and patient information.

### Technical aspects and radiographic device

All X-ray images were obtained with a mobile X-ray unit (Mobilett XP digital, Siemens Healthineers) using dedicated X-ray exposure detector cassettes: Fujifilm FCR IP Cassette, Type CC, 18 × 24 cm as well as Fujifilm FCR IP Cassette, Type CC, 24 × 30 cm (Fujifilm) depending on the size of the child (Supplementary Fig. 1). The images were read out on a Philips PCR Eleva Corado (Philips Healthcare) (Supplementary Fig. 2). All devices used have European approval and conformité Européenne marking. The total filtering was 4.4 mmAl + 0.1 mmCu. Whenever possible incubator trays were not in use and the detector was placed behind the region of interest to carry out the examination. However, in some cases, incubator trays had to be used, especially in very sick children who could not be moved for the examination.

### Measurement of the DAP and calculation of the estimated dose (ED)

The DAP in mGy*cm² could be obtained from GE Centricity Universal Viewer in 3067 of 4168 images. The missing values were determined by using simple linear regression (DAP vs body weight) in GraphPad Prism 9.0.0 (GraphPad Software, San Diego, CA, USA), where X is the body weight at the time of imaging:

- Thoracic X-ray: *Y* = 10^(1.008 × log10 (X) − 4.158)^;
- Abdominal X-ray: *Y* = 10^(1.201 × log10 (X) − 4.673)^;
- Combined thoracic/abdominal X-ray: *Y* = 10^(0.861 × log10 (X) − 3.542)^.

These calculated values made it possible to determine the cumulative DAP.

Different tissues have different sensitivities to ionising radiation, so it is important to determine the effective dose (ED). Its calculation uses specific weighting factors for individual tissues and organs and can contribute to the risk assessment of X-ray imaging. Although there is controversy regarding the use of the ED [10], it has been used repeatedly in adults as well as in paediatric radiology [11–25]. Of note, the ED is only an estimate, and there are many different methods to determine it. All methods utilise conversion coefficients with which the ED can be estimated from the input variables, namely DAP, the entrance skin dose or air kerma. We used the method developed by Elbakri et al [11]. Conversion coefficients (µSv/mGy*cm²) are determined by using the tube voltage, weight class (500–6000 g), the type of image (anteroposterior thorax/abdomen) and the X-ray filter (3 mmAl + 0.1 mmCu) [11]. With the approach, the ED could be estimated from the DAP. Because there were no coefficients for the combined thoracic/abdominal X-rays, the DAP for these images was multiplied by the averaged coefficient for the thoracic and abdominal X-rays.

### Risk estimation

The International Commission on Radiological Protection (ICRP) Publication 60 [26] includes risk assessment factors (2.8–13 × 10^−2 ^Sv^−1^) that allow estimation of the radiation-induced risk of cancer—especially leukaemia, but also other types of cancer—in the first decade of life. We used the upper value of 13 × 10^−2 ^Sv^−1^. The ICRP released Publication 147 in 2021 [27], which clarifies that the estimated risk for the 0–9-year age group should be twice as much as the estimated risk factors of the ICRP nominal coefficients published in ICRP Publication 60. The nominal risk coefficient for all age groups is 6 × 10^−2 ^Sv^−1^. Of note, the risk assessment factor that we used, 13 × 10^−2^ Sv^−1^, is in the upper reference range and thus still applies. Because the ED, cumulative ED and risk are estimates, they are given in absolute numbers and also grouped according to risk levels: ‘negligible’ (< 0.1 mSv), ‘minimal’ (0.1–1 mSv), ‘very low’ (1–10 mSv) and ‘low’ (10–100 mSv) [10].

### Data analysis

We used Microsoft Excel (Microsoft), GraphPad Prism 9.0.0 (GraphPad Software), BiAS for Windows (Epsilon) and STATA IC 12 (StataCorp LLC, College Station, TX, USA) for statistical analysis. We report descriptive statistics in tables. We used the Kruskal–Wallis test followed by Dunn’s test to assess the non-normally distributed datasets. Quantitative features that correlate with one another could be mapped as a pair of values using a point cloud. We present the distribution of the data with box and whisker plots. We used the Yates–Cochrane test to assess trends. We considered *p* < 0.05 to indicate a statistically significant difference. In the figures, asterisks indicate the level of significance according to the American Psychological Association (APA) style: ns (0.12), * (0.033), ** (0.002) and *** (< 0.001). Our study is one of the largest studies on this subject (with 1307 patients), so the sample size is sufficient.

## Results

### Patients and medical conditions

We divided the 1307 patients (mean age 241.1 ± 35.45 days) into five classes based on birth weight:

- Group 1: < 500 g;
- Group 2: extremely low birth weight, ≥ 500 g to < 1000 g;
- Group 3: very low birth weight (VLBW), ≥ 1000 g to < 1500 g;
- Group 4: low birth weight, ≥ 1500 g to < 2500 g; and
- Group 5: normal birth weight, ≥ 2500 g.

Table 1 shows the characteristics of these patients as well as their medical conditions. The infants were hospitalised for an average of 30.4 days (median 16, range 1–255 days). The length of stay (LOS) correlated negatively with birth weight and gestational age. Four hundred ninety-four of the patients were intubated for at least 1 day. The patients who were intubated received mechanical ventilation for an average of 9.4 ± 16 days. Respiratory distress syndrome (RDS) was diagnosed in 74.2% of the complete cohort. A central catheter was placed in 39.5% of the patients.

**Table 1:** Patients characteristics and their medical conditions in relation to the 5 birth weight categories

Characteristic		5 birth weight categories in g				
	Number of patients (*n* = 1307) *n* (%)	< 500 g	≥ 500 g bis < 1000 g	≥ 1000 g bis < 1500 g	≥ 1500 g bis < 2500 g	≥ 2500 g
		17 (1.3)	175 (13.4)	197 (15.1)	301 (23)	617 (47.2)
LOS days	Median (range)	129 (195)	78 (255)	54 (166)	24 (194)	8 (96)
Birth size cm	Mean (SD)	29.1 (5.1)	33.6 (3.9)	38.6 (3.0)	44.2 (3.1)	51.5 (3.1)
Diagnosis *n* (%)						
RDS		15 (88)	166 (95)	181 (92)	239 (79.4)	367 (59.5)
BPD		12 (71)	68 (39)	8 (4)	2 (0.7)	3 (0.5)
NEC		5 (29)	24 (14)	6 (3)	7 (2.3)	0
Therapeutic procedures						
Central line	*n* (%)	15 (88.2)	149 (85.1)	125 (63.5)	79 (26.2)	149 (24.1)
Length of intubation in days (*n* = 494)	Mean (SD)	41.8 (50.0)	13.8 (14.0)	7.1 (12.8)	4.5 (6.0)	6.5 (11.7)
Length of non-invasive ventilation in days (*n* = 677)	Mean (SD)	35.1 (22.0)	23.0 (19.0)	6.7 (6.5)	2.3 (2.1)	2.4 (3.1)

*LOS* length of stay, *RDS* respiratory distress syndrome, *BPD* bronchopulmonary displasia, *NEC* necrotising enterocolitis

**Fig. 1:**
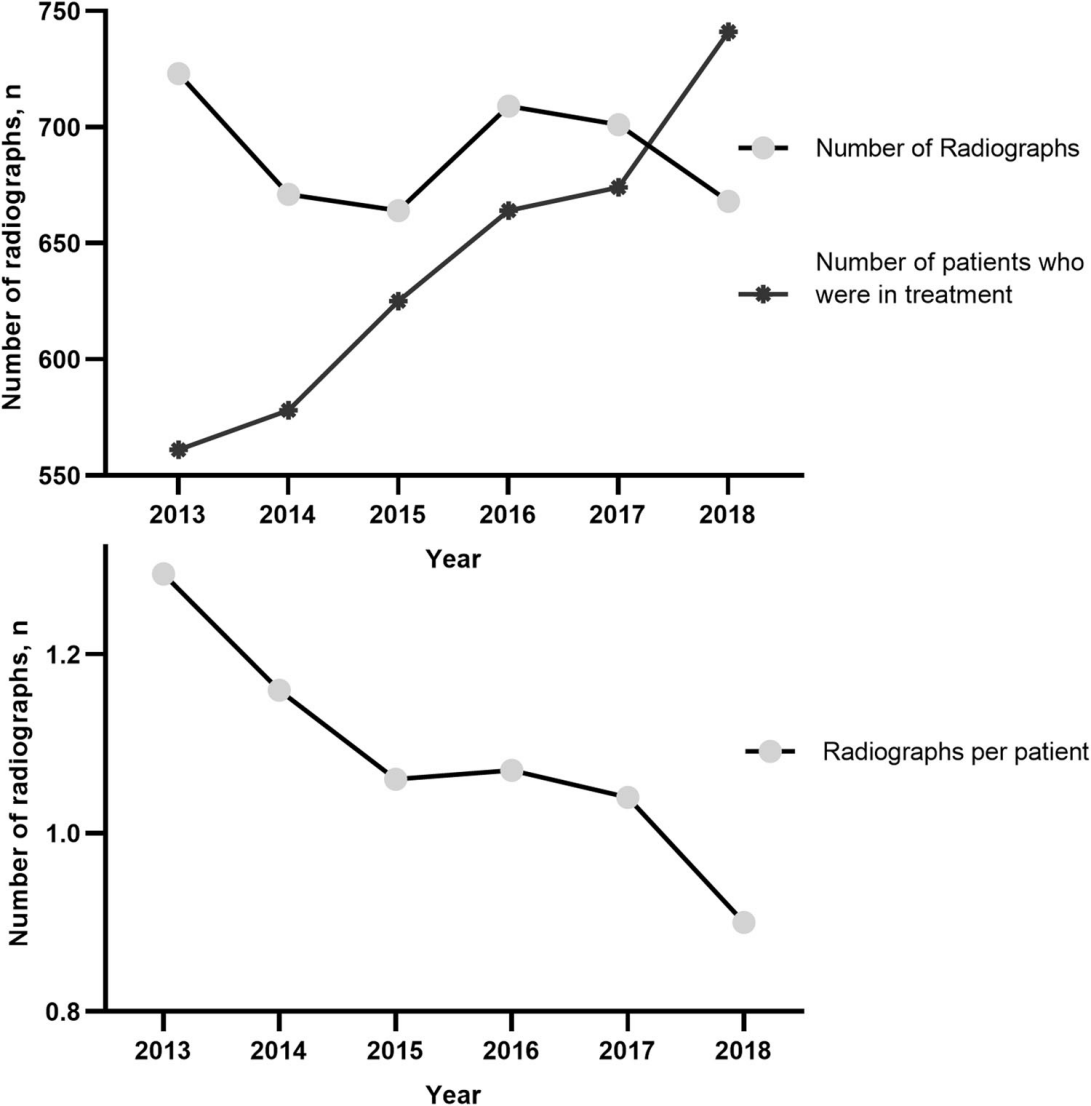
The bottom panel shows the number of radiographs per patient. The top panel shows the number of radiographs and the number of treated patients per year

The number, frequency and type of radiographs: from a total cohort of 3843 patients who were treated in the given timeframe, 1307 (34%) received at least one X-ray. The average number of X-rays per patient decreased continuously over the years, with 0.9 images per patient and stay in 2018 (Fig. 1).

**Fig. 2:**
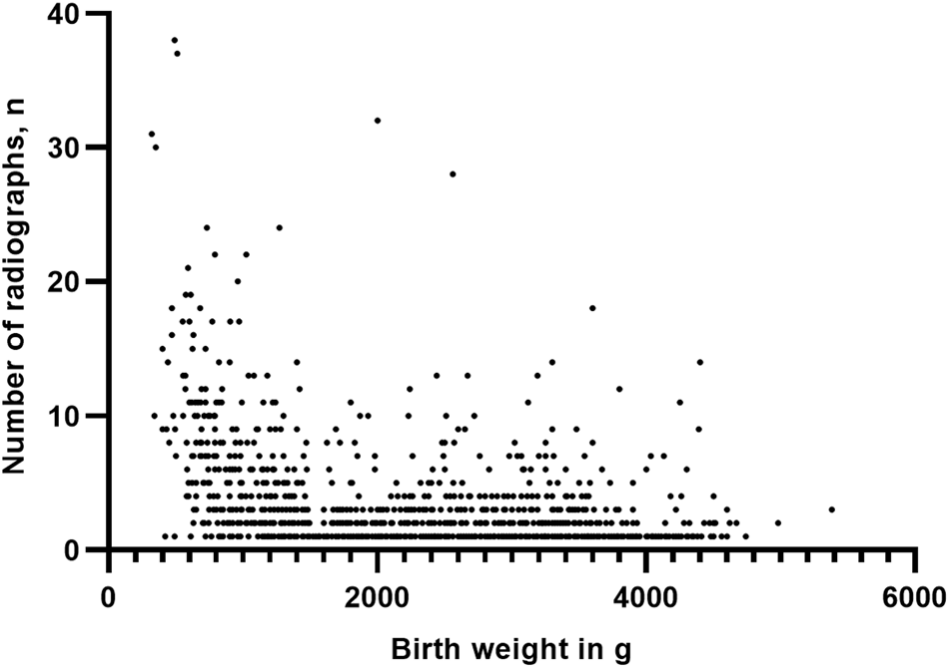
The correlation between the number of radiographs and birth weight. The number of radiographs correlates significantly with birth weight (*r* = −0.43, *p* < 0.001, *n* = 1307)

The mean number of X-rays per patient in the study population (*n* = 1307) was 3.19 (range 1–38). The mean number of X-rays decreased as birth weight increased (Fig. 2).

**Fig. 3:**
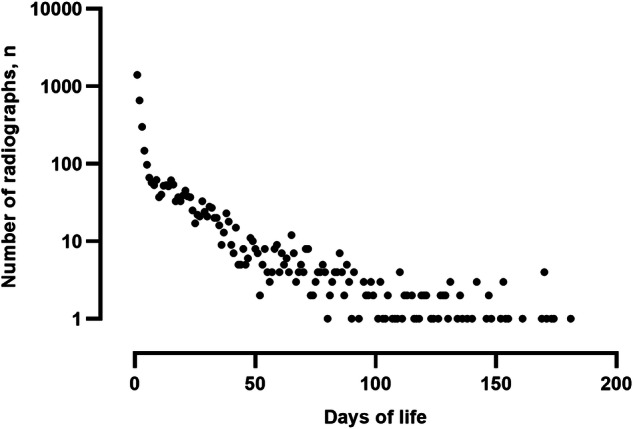
The correlation between the number of radiographs and the days of life (logarithmic representation). The number of radiographs correlates significantly with days of life (*r* = −0.9043, *p* < 0.001, *n* = 181)

We found that 614 (47%) of the patients received only one X-ray. However, 62 (4.8%) of the patients received 11–20 X-rays. As age increased, the number of X-rays decreased continuously (Fig. 3). Most images (*n* = 1404) were taken immediately after birth on the first day of life.

More combined thoracic/abdominal X-rays were acquired with a lower birth weight, and more thoracic X-rays were acquired with a higher birth weight. The type of radiograph was related to birth weight (Yates–Cochrane test for trend: chi-square = 25.04, *p* < 0.001, *n* = 4090) and gestational age (chi-square = 25.37, *p* < 0.001, *n* = 4090). Immature patients often need medical support immediately after birth, for example, the placement of an umbilical vein or umbilical artery catheter. These interventions are evaluated with combined thoracic/abdominal X-rays or, in rare cases, with only additional abdominal X-rays, which explains the slightly higher number of abdominal images in the lower birth weight groups.

### The DAP, current-time product, tube voltage and ED for different types of X-rays

**Fig. 4:**
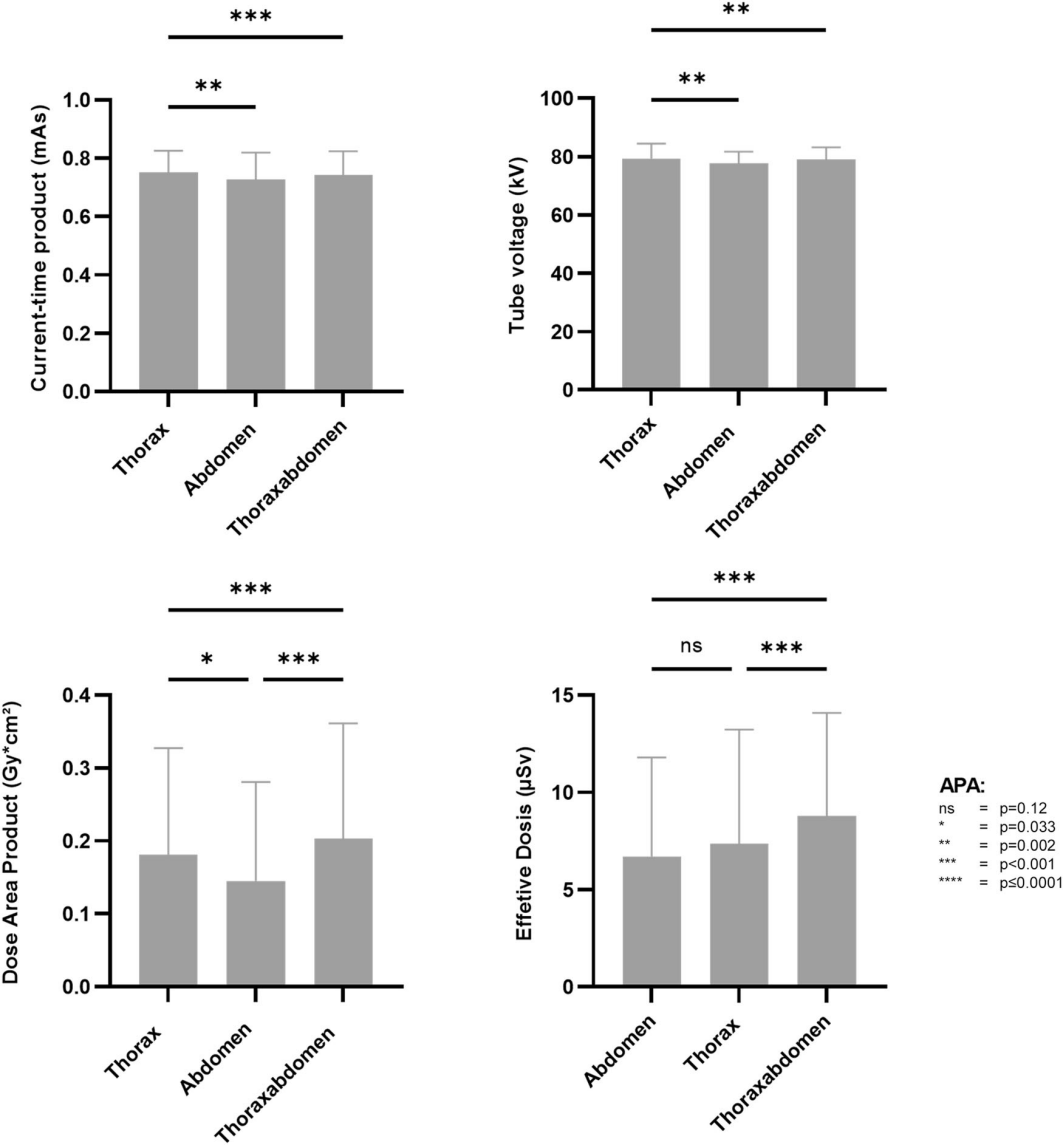
The current-time product (mAs), tube voltage (kV), dose area product (DAP, µGy*m^2^) and estimated dose (ED, µSv) for each type of radiograph. The data are presented in box and whisker plot

The average DAP was 1.9 mGy*cm² per radiograph. It was highest for the combined thoracic/abdominal X-rays, with a mean of 2 ± 1.6 mGy*cm² (*p* < 0.001) (Fig. 4). The ED was 8.8 ± 5.3 µSv (*p* < 0.001). Overall, the diagnostic guidance for a thoracic X-ray in preterm infants is 3 mGy*cm^2^, and thus the average in this study is well within the diagnostic guidelines [28].

### The DAP, current-time product, tube voltage and ED in relation to birth weight

The DAP and ED were increased in patients whose birth weight was ≥ 2500 g (*p* < 0.001). Patients whose birth weight was < 500 g also showed an elevated ED, but the DAP per image was low (Table 2).

**Table 2:** Tube voltage, DAP, Current-time product in relation to the type of X-ray

Birth weight	Tube voltage (kV). Mean (SD)	Current-time product (mAs). Mean (SD)	DAP (mGy*cm²) Mean (SD)	ED (µSv) Mean (SD)
< 500 g	76.42 (2.89)	0.69 (0.06)	1 (1)	8.3 (8.4)
≥ 500 g bis < 1000 g	76.45 (2.83)	0.71 (0.11)	1.1 (1)	7.4 (6.3)
≥ 1000 g bis < 1500 g	77.45 (2.84)	0.73 (0.06)	1.4 (1.1)	6.7 (5.4)
≥ 1500 g bis < 2500 g	78.61 (5.38)	0.77 (0.09)	1.9 (1.2)	6.8 (4.6)
≥ 2500 g	83.27 (3.84)	0.79 (0.07)	3.1 (1.6)	9.2 (4.4)

*DAP* dose area product, *ED* effective dose

### The cumulative DAP, ED and estimated risk in relation to birth weight

**Fig. 5:**
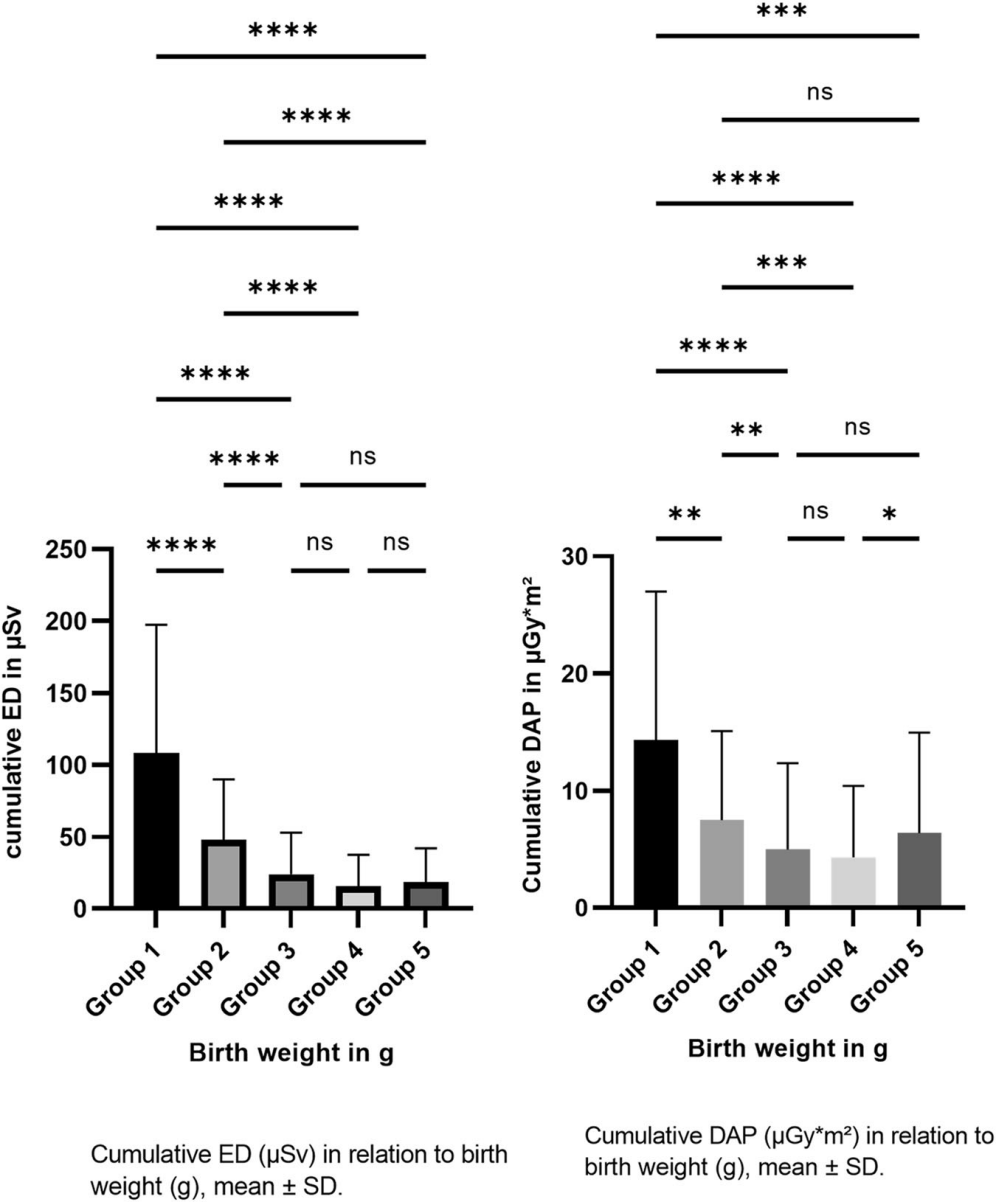
The relationship between the cumulative estimated dose (ED, µSv) and the cumulative dose area product (DAP, µGy*m^2^) and birth weight (g)

The cumulative DAP, the ED per hospital stay and the estimated risk of developing cancer after certain radiation exposure decreased as birth weight increased (*p* < 0.01) (Table 3 and Fig. 5). The cumulative DAP per stay of a premature or sick newborn peaked in patients with a birth weight of < 500 g (14.3 mGy*cm² per patient). The maximum cumulative DAP in the < 500 g birth weight group was 45 mGy*cm² in a preterm infant born at gestational week 24 + 3 with a birth weight of 490 g. This patient was hospitalised for 194 days and received 38 X-rays during this time (the patient had many medical conditions that required monitoring by X-ray). Patients with a birth weight of ≥ 2500 g were hospitalised for a mean number of 8 days, about 16 days shorter than those with a birth weight of ≥ 1500 g to < 2500 g. However, this cohort had the highest mean DAP per image at 3.1 mGy*cm², a phenomenon that is explained by the ‘exposure area’, which increases in smaller infants. This explains why the mean cumulative DAP for this cohort increased to 6.4 mGy*cm².

**Table 3:** LOS, Number of radiographs, Cumulative DAP, ED and risk estimation in relation to the birth weight

Birth weight	LOS. median (range)	Number of radiographs. Mean (SD)	Cumulative DAP (mGy*cm²) Mean (SD)	Cumulative ED (µSv). Mean (SD)	Cumulative risk (*10^−6^) by (13*10^−2 ^Sv^−1^)
< 500 g	129 (195)	13.9 (10.3)	14.3 (12.7)	108.2 (89.3)	14
≥ 500 g bis < 1000 g	78 (255)	7.1 (5.4)	7.5 (7.6)	47.7 (42.2)	6.2
≥ 1000 g bis < 1500 g	54 (166)	3.6 (3.6)	5 (7.4)	24 (28.8)	3.1
≥ 1500 g bis < 2500 g	24 (194)	2.3 (2.7)	4.3 (6.1)	15.8 (21.6)	2.1
≥ 2500 g	8 (96)	2.12 (2.4)	6.4 (8.6)	18.5 (23.6)	2.4

*DAP* dose area product, *ED* effective dose, *LOS* length of stay

**Fig. 6:**
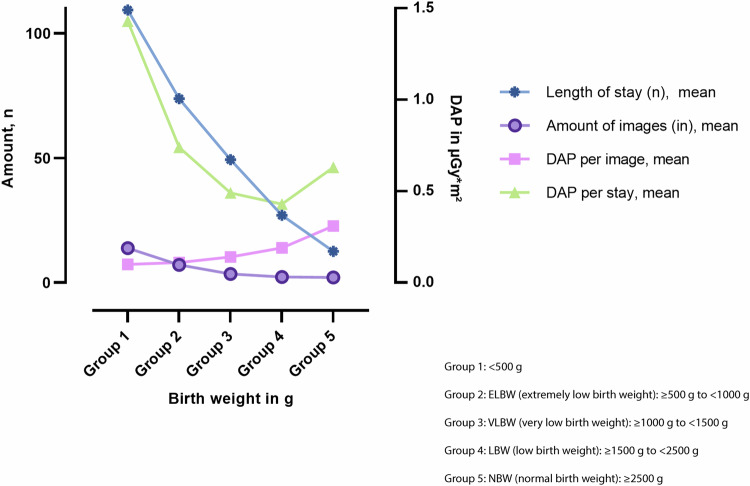
The relationship between the length of stay, number of images and dose area product (DAP, per image/stay) and birth weight (g)

The X-rays taken in the < 500 g birth weight group resulted in a cumulative estimated risk of 14 × 10^−6^. This means that out of 1,000,000 patients examined, 14 could develop a malignant disease. Thus, the estimated risk can be classified as ‘minimal’, while it was only ‘negligible’ in the other birth weight groups according to the definition of Martin et al [10] (Table 3). The relative risk model is used in many international societies such as ‘Image Wisely’ by the American College of Radiology and the ‘iGuide’ from the European Society of Radiology [29, 30]. We evaluated this connection in detail (Fig. 6). The cumulative DAP per stay initially decreased (green) because the patients had a shorter stay (blue) and received fewer radiographs (purple). However, for patients with a birth weight of ≥ 2500 g, the cumulative DAP increased slightly due to the increase in DAP per image.

## Discussion

The number of X-ray examinations: we showed a correlation between the number of X-rays and prematurity, birth weight and LOS. We compared this finding with six different studies published from 1996 to 2014. Overall, the number of X-ray examinations has decreased over the last decades. For the weight category < 750 g, there was a mean of 31 examinations in 1996 [23], 26 in 2002 [22] and 19.5 in 2006 [25]. Puch-Kapst et al [20] reported a mean of 11 examinations in 2009. In our study, we found 10.1 examinations in the < 750 g birth weight group. For patients with a birth weight of < 1000 g, there was a mean of 9.6 X-ray examinations in 2008 [31], 15.4 in 2014 [32] and 7.7 in our study. Our evaluation in a level 1 NICU showed a lower average number of X-rays compared with studies from other countries, but it appears to be in line with the current medical standards and literature [33].

### Tube voltage, current-time product, DAP, ED, and estimated risk in our study compared with the literature

Many researchers have performed a risk assessment following X-ray examinations [12, 20–24, 32, 34]. The estimated risk values vary from 1 × 10^−6^ for a thoracic image [21] to 5.2 × 10^−6^ for a combined thoracic/abdominal image [23]. Even the overall concept of calculating the ED has been discussed [35, 36]. This discussion has included the factors that influence radiation exposure and sensitivity to radiation exposure, but reducing exposure to ionising radiation remains an overarching goal for the medical use of X-ray imaging [35, 36]. An important issue is weighing the benefits of X-ray imaging with the risk of developing cancer due to X-ray exposure. In the current literature, the overall risk of developing cancer due to X-ray imaging remains ‘negligible’ [37]. Our calculated risk factor is only 1.01 × 10^−6^ regarding the risk of cancer after a single diagnostic X-ray. Given the diagnostic and therapeutic benefits of X-ray imaging, this remains an acceptable risk.

In our analysis, the DAP was 1.8 ± 1.5, 1.5 ± 1.4 and 2 ± 1.6 mGy*cm² for thoracic, abdominal and thoracic/abdominal X-ray examinations, respectively. In 2001, Jones et al [19] reported the following mean values: 8.3 mGy*cm² for thoracic X-rays, 11.5 mGy*cm² for abdominal X-rays and 18.7 mGy*cm² for thoracic/abdominal X-rays, which are well above our values. In 1996, McDonald et al [38] determined a mean DAP of 15 mGy*cm² for thoracic X-rays and 19 mGy*cm² for abdominal X-rays. In 2014, Dabin et al [32] determined a DAP of 4.1–7.2 mGy*cm². This study is more recent to ours and thus closer to our values. Overall, there is a large variance in the reported DAP; our values remain very low compared with the literature.

The current literature [21, 39–41] as well as national [42] and international [43] guidelines recommend increasing the tube voltage with a shorter exposure time and the use of X-ray filters to reduce the radiation dose while maintaining image quality. Duggan et al [44] reported a 9% reduction in radiation exposure if the tube voltage could be increased from 50 to 60 kVp. However, there are X-ray units that cannot generate the short exposure times required for the high recording voltage. Older X-ray machines, which were used in studies in the late 1990s and early 2000s, were not able to work under these conditions and with these settings. Table 4 gives a detailed overview of the literature as well as the differences between the tube voltage and the current-time product. However, in CT imaging “low kV” imaging is gaining more and more importance, if this will be important for conventional X-ray imaging is not clear up to now [45].

**Table 4:** Tube voltage, current-time product, DAP, entrance skin dose, ED, estimated risk in relation to the type of radiograph

Author	Type of radiograph	Tube voltage (kV) Mean (SD)	Current-time product (mAs) Mean (SD)	DAP (mGy*cm²) Mean (SD)	Entrance skin dose (µGy) Mean (SD)	ED (µSv) Mean (SD)	Risk estimation (*10-6 examinations) (ED*13*10^−2^)
This study	Thorax	79.4 (5)	0.75 (0.07)	1.8 (1.5)	-	7.4 (5.9)	1
	Abdomen	77.7 (4)	0.73 (0.09)	1.5 (1.4)	-	6.7 (5.1)	0.9
	Thorax/abdomen	79 (4.2)	0.74 (0.08)	2 (1.6)	-	8.8 (5.3)	1.2
Jones et al [15]	Thorax	62	2	8.3	56.7 (0.2)	15.4	2
	Abdomen	62	2.5	11.5	73.6 (0.2)	21.9	2.8
	Thorax/abdomen	62	2.5	18.7	71.5 (0.2)	35.5	4.6
McDonald et al [27]	Thorax	62	8.6	15	60	-	-
	Abdomen	59	3.6	19	110	-	-
	Thorax/abdomen	-	-	-	-	-	-
Mooney et al [30] *(value) = after use of higher filtration	Thorax	55	-	12 (7)*	60 (36)	-	-
	Abdomen	62	-	29 (20)	170 (120)	-	-
	Thorax/abdomen	-	-	-	-	-	-
Puch- Kapst et al [16]	Thorax	-	-	-	-	14.4	1.9
	Abdomen	-	-	-	-	17.8	2.4
	Thorax/abdomen	-	-	-	-	23.8	3.2
Armpilia et al [17]	Thorax	53.1	2	4.3	36	7.8	0.3–1.3
	Abdomen	53	2.1	4.4	39	10.2	0.3–1.3
	Thorax/abdomen	52.2	2	5.5	35	9.2	-
Olgar et al [20]	Thorax	49	1.9	-	67	15	2
	Abdomen	48	2	-	65	22	2.9
	Thorax/abdomen	-	-	-	-	-	-
Dabin et al [24]	Thorax	-	-	4.1–7.2	41–46	-	-
	Abdomen	-	-	-	-	-	-
	Thorax/abdomen	-	-	-	47	-	-
Ono K. et al [18]	Thorax	55	1.2	-	21	10–20	-
	Abdomen	55	1.2	-	10–20	10–30	-
	Thorax/abdomen	55	1.2	-	10–20	20–40	-
Wilson-Costello et al [19]	Thorax	-	-	-	-	10–20	2.6
	Abdomen	-	-	-	-	10–30	3.9
	Thorax/abdomen	-	-	-	-	20–40	5.2
Makri et al [8]	Thorax	50 (2)	1.5 (0.3)	-	44 (16)	10 (3.7)	1.7
	Abdomen	-	-	-	-	-	-
	Thorax/abdomen	49 (2)	1.5 (0.3)	-	43 (19)	14.7 (7.6)	2.9
McParland et al [28]	Thorax	52–60	0.8	-	20 (3.2)	-	14
	Abdomen	52–60	0.8	-	20 (4.2)	-	17.1
	Thorax/abdomen	-	-	-	19 (2.7)	-	21.6

### The cumulative ED and risk assessment in the literature in relation to birth weight

Patients in the < 500 g birth weight group received a larger cumulative ED during their hospital stay (108 ± 89 µSv), resulting in an estimated risk of 14 × 10^−6^ of developing cancer. The risk can be classified as ‘minimal’. The highest cumulative ED was 342 µSv after 30 examinations of a patient with a birth weight of 350 g; the estimated risk of 44 × 10^−6^ can also be classified as ‘minimal’. The cumulative estimated risk in all other weight classes was ‘negligible’ [37]. A 2004 study from Kuwait reported a risk of 9–114 × 10^−6^ in a patient who received 25 X-rays during his/her stay [18]. In a study with 194 patients with a VLBW who received a mean of four X-rays (range 1–62), the cumulative risk was 17.5 × 10^−6^ per patient [20]. Makri et al [12] determined a cumulative risk of 1 × 10^−4^. In 1998, Sutton et al [34] evaluated the data from 55 patients with a VLBW and reported a risk of 9.1 × 10^−6^. A 1994 study with 119 newborns with a mean number of 5.3 X-rays per patient determined a risk of 5.2 × 10^−6^ [46].

### Alternative imaging strategies

Conventional X-ray for examination of the lungs remains the preferred examination method in most clinical settings, especially in acute settings such as intensive care units. Nevertheless, there are alternatives that have been analysed and evaluated in many studies and that have emerged in recent years [1–4]. In their 2021 review, Sandig et al [47] discussed sonography as an alternative examination method for pneumothorax. The overall sensitivity of sonography was higher than X-ray examinations, and the specificity was similar between the two imaging modalities.

### Limitations

First, the excluded patients who died at less than 14 days (*n* = 32) and those whose documentation was incomplete (*n* = 17) were mostly premature with high morbidity and thus had a high pre-test probability for many examinations. Second, this was a retrospective study, so we had to face the issues inherent to analysing retrospective data. The follow-up of the X-ray examined patients over decades was and is still not possible because of budget limitations, and funding from the government is often not available for these long time periods. Third, the overall methodology using DAP as well as conversion coefficients for correlation between radiation exposure and the ED continues to be discussed in the radiology community and beyond. There are risk estimates that still cannot be included in detail and some uncertainty remains. However, it remains a good approximate measure of radiation risk and in daily practice, it provides the user with an adequate overview of the exposure in recent years. Fourth, we only included thoracic, abdominal or combined radiographs. We did not include other examinations that use ionising radiation such as fluoroscopy, CT, interventional radiology or radiographs of the extremities. Overall, this would be an interesting topic for further investigation, especially if the decrease in the use of thoracic and abdominal X-ray imaging is transferable to other examinations that use ionising radiation.

## Conclusion

In conclusion, we confirmed a correlation between prematurity and radiation exposure. However, our data do not indicate that early diagnostic imaging leads to a long-lasting health risk regarding the development of cancer. The benefits of X-ray diagnostics in premature and full-term infants, especially when positioning catheters or tubes and confirming or ruling out probable life-threatening diseases, outweigh the minimal estimated risk of radiation exposure. Nevertheless, according to the As Low As Reasonably Achievable principle, there are still aspects to improve. In the future, particular attention should be given to patients with a birth weight of < 500 g, as well as to the combined thoracic/abdominal images. Alternatives such as sonography should be used, and training and quality management must be continued.

Overall, our main results are as follows:

- Half (*n* = 614) of all patients received only one X-ray. Most of them (*n* = 539) had a birth weight of ≥ 1500 g.
- This radiation risk can be classified as ‘minimal’ for patients with a birth weight of < 500 g and as ‘negligible’ for all other birth weight groups.

## Supplementary information

ELECTRONIC SUPPLEMENTARY MATERIAL

## Abbreviations

CT: Computed tomography
DAP: Dose area product
ED: Effective dose
ICRP: International Commission on Radiological Protection
LOS: Length of stay
NICU: Neonatal intensive care unit
RDS: Respiratory distress syndrome
Sv: Sievert
VLBW: Very-low birth weight
