# Supplementary material for: Radiation exposure and estimated risk of radiation-induced cancer from thoracic and abdominal radiographs in 1307 neonates
Source: Eur Radiol. 2024 Jul 16;35(1):297–308. doi: 10.1007/s00330-024-10942-x (PMC11632034; doi:10.1007/s00330-024-10942-x)
Supplement: Supplementary file 1 — ELECTRONIC SUPPLEMENTARY MATERIAL [file 330_2024_10942_MOESM1_ESM.pdf]

Radiation exposure and estimated risk of radiation-induced cancer from thoracic and abdominal radiographs in 1307 neonates

ELECTRONIC SUPPLEMENTARY MATERIAL

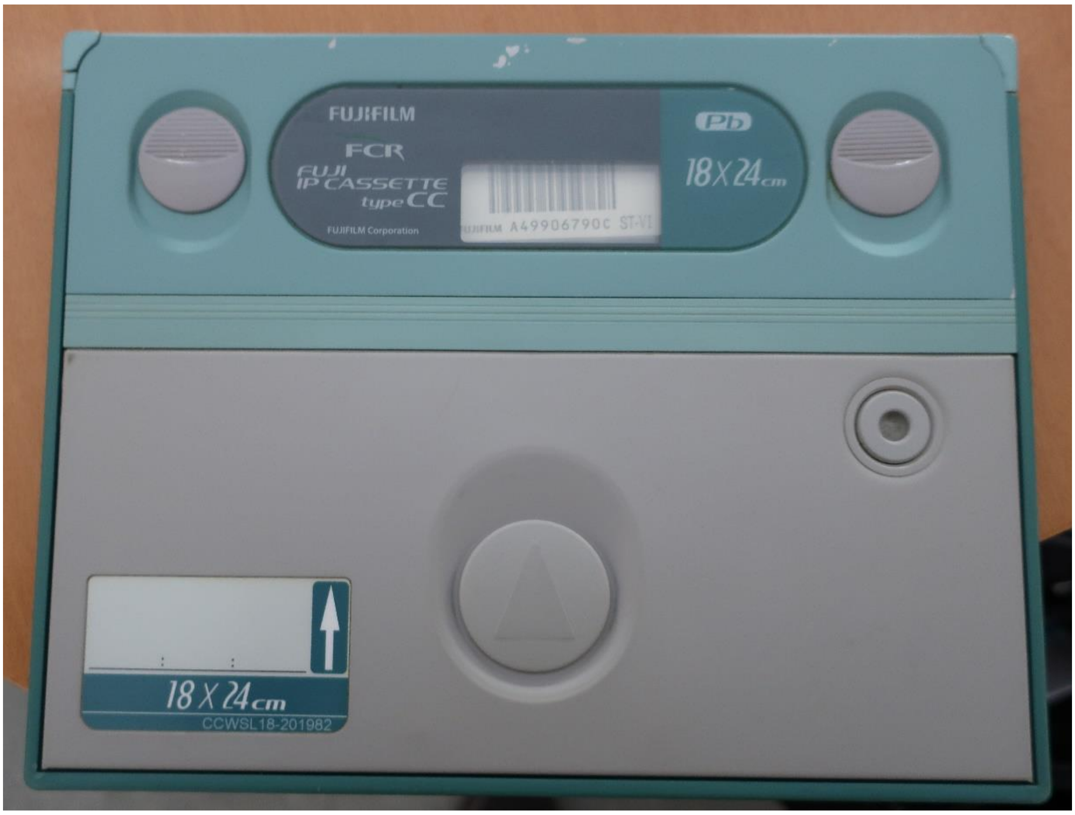

Suppl. Fig. 1: Fujifilm 18 x 24 cm cassette for imaging on the NICU.

Eur Radiol (2024) Weiß D, Beeres M, Rochwalsky U, Vogl TJ, Schlößer R.

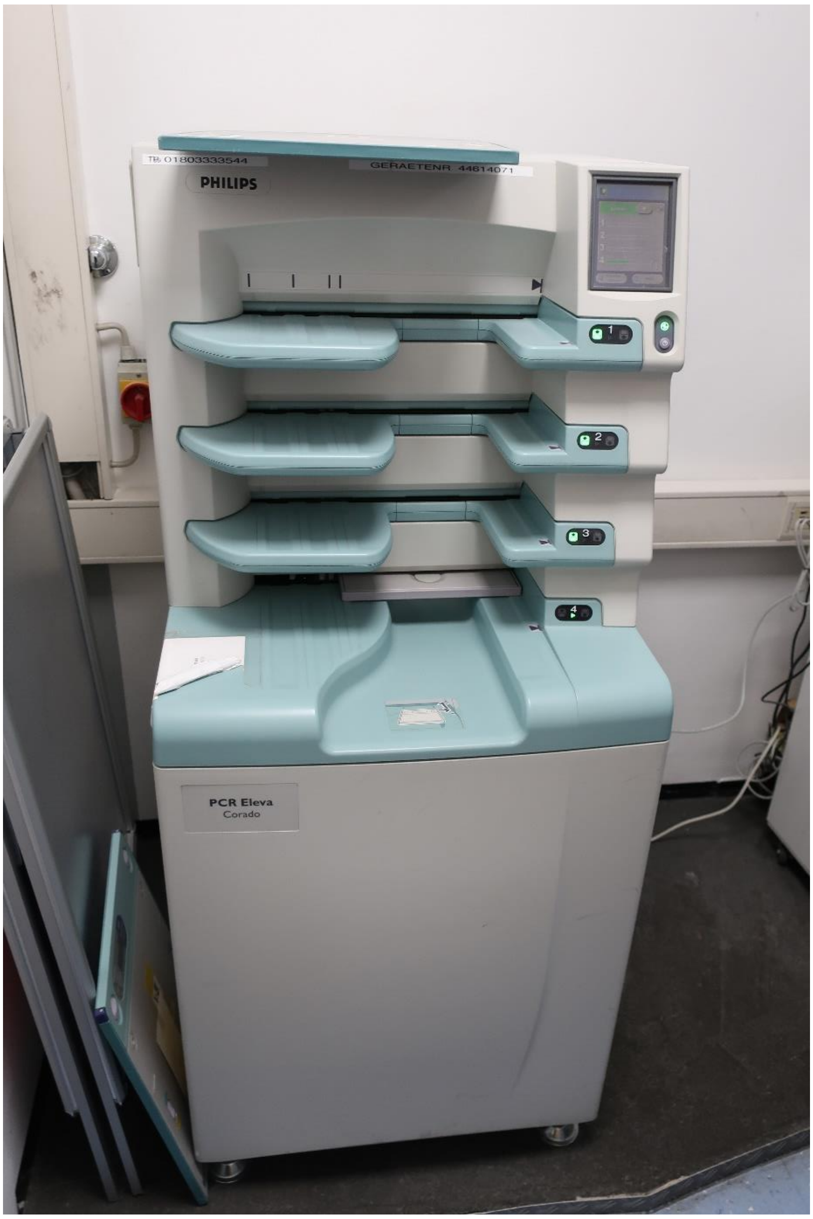

Suppl. Fig. 2: PCR Eleva Corado Read-Out System
